# Ten simple rules for creating a sense of belonging in your research group

**DOI:** 10.1371/journal.pcbi.1010688

**Published:** 2022-12-08

**Authors:** Nidia Ruedas-Gracia, Crystal M. Botham, Amber R. Moore, Courtney Peña

**Affiliations:** 1 College of Education, University of Illinois at Urbana-Champaign, Champaign, Illinois, United States of America; 2 Stanford Biosciences Grant Writing Academy, Stanford University, Stanford, California, United States of America

## Introduction

Across academic fields and disciplines, we’re hearing more and more buzz around the importance of sense of belonging. However, two important elements are missing from these conversations. First, we need to have a grounded understanding of what sense of belonging is, and how it can be cultivated. Secondly, these conversations tend to apply to a typical classroom context but what about the sense of belonging in research and lab spaces?

Sense of belonging is generally defined as the experience of positive personal relationships with others in a given environment [1]. It is an important predictor of well-being [2] and retention in science, technology, engineering, and mathematics (STEM) [3]. There are many benefits associated with developing a strong sense of belonging including improvements in academic performance, mental health, self-esteem, sense of purpose, and connectedness [4,5,6,7]. On the other hand, there are many significant consequences of having a low sense of belonging such as increased risk of stress, anxiety, depression, health problems, feelings of loneliness, rejection, and low self-esteem [2,5,8,9]. Creating environments that are conducive to well-being and belonging not only serve the principal investigator (PI) and the trainee, but also has the potential to increase research integrity, increase and retain the diversity among scientific leaders, and transform academia.

Research labs, whether wet or dry, academic or field-based, are important learning environments where many graduate students, trainees, technicians, and postdocs spend a significant amount of time. Yet, despite the amount of time spent in the lab, trainees do not always feel that they belong there [10]. This paper addresses this lack of belonging by proposing ten simple rules for creating a sense of belonging in your research group. These rules (S1 Fig) are grounded in evidence-based practices that have increased the retention of systemically and historically excluded groups [11,12,13,14,15], and improved the mental health crisis in graduate and postdoc education, as belonging is strongly tied to mental health and well-being [16,17,18,19,20]. Because an individual’s sense of belonging can change and evolve over time, PIs and senior lab members can use these rules to establish and maintain a culture where individuals feel valued, heard, and appreciated. Sense of belonging impacts workplace performance [21]; therefore, having a strong sense of belonging within your research group positions lab members to not only have a higher quality of life, but also produce higher-quality research and successfully progress in their research careers. This model can then be emulated by emerging scientists who will become leaders and effective mentors themselves, allowing the cycle to repeat. In this article, we draw from research on sense of belonging and educational psychology to inform best practices for fostering a sense of belonging in the lab. This paper is informed by our experiences as former lab members (graduate students/postdocs), current PIs of research labs, and education researchers. This paper will be most useful for those leading labs (e.g., PIs), or lab members who want to improve a sense of belonging within the lab environment.

### Rule 1: Reflect on belonging (and repeat)

Self-reflection is an important first step in identifying what kind of culture and environment you want to foster in your research group. Whether you are new or well established, managing a small group or a large one, it is never too late (or early!) to begin thinking about belonging. You can begin the reflection process by thinking about a time during your early career when you felt like you belonged. Ask yourself, what was the environment like? How did others interact with you that made you feel like you were important to the people around you? For example, was there a specific bonding experience like a potluck where you shared a cultural dish with your lab mates? Was there a symbolic item that brought everyone together like a lab t-shirt or website that included everyone’s name and/or image? Were your accomplishments celebrated by your lab in a way that made you feel recognized and validated? These are examples and what you do in your lab should cater to the interests and identities of the team. Use these experiences as a motivator to emulate the types of experiences you want in your research group.

Next, think about some experiences when you felt like you did not belong. Ask, what was going on that made you feel this way? How were others interacting (or not interacting) with you? How did those experiences make you feel? These might be unpleasant memories to revisit, but they can be crucial in identifying the types of practices that you do not want to replicate in your lab.

Then, reflect on how you engage and communicate with members in your lab. Ask yourself the following questions: What do you do to show that you’re invested in their success? How do you talk to them? How do you provide feedback? How do you support their career growth? At your group meetings, who speaks the most? Who interrupts the most? As a leader, it’s important to acknowledge the power dynamics involved and ensure that the feedback shared in your group is constructive and supportive, especially for your developing scientists who are still building their confidence.

It is common to model the behavior you witnessed throughout your career, even when harmful, because breaking with tradition and changing the culture can be awkward and difficult. But, more likely than not, your research group is already quite familiar with seeking challenges and taking risks to reimagine what is possible and improve the human experience. Why not apply this approach to belonging?

When in doubt, return to the definition. Sense of belonging is the experience of *positive personal involvement* in a system or community so that *persons feel themselves to be an integral part of that system* [22]. Use the definition to guide conversations with your lab. You can pose the following questions to get a sense of how people are feeling: Does everyone feel positively involved in the lab? Do they feel like they are an important part of the lab? Psychological science tells us that feelings are not only important, but also serve as strong predictors of human behavior and performance [23]. Provide opportunities to hear how people are feeling so that you can make adjustments as needed. Revisiting and reflecting on these guiding questions are the first and most important steps you can take towards improving sense of belonging in your lab.

### Rule 2: Be mindful of names, pronouns, and diverse identities

Don’t underestimate the importance of knowing names and how to pronounce them correctly [24]. No matter what your cultural or linguistic background is, it is likely (and should be the case) that you will have people in your lab from identity groups (e.g., race, ethnicity, gender, sexual orientation, nationality) that are different from yours. This difference might result in a gap between your cultural and linguistic norms and those of your lab members. Education research tells us that mispronouncing a student’s name can be harmful and even painful for a student [25]. This phenomenon extends to professional settings as well and can signal to a group about who belongs and who does not. Taking the time to ask people how they want to be addressed, including name, title, and pronouns, can make a big difference in whether or not an individual feels like they belong in your lab.

If you’re not sure if you are pronouncing a name correctly, simply ask: “Did I say your name correctly?” [26]. Additionally, your team can use NameCoach [27] to have audio name pronunciations embedded online (e.g., lab website, email signatures) to learn and remember how to say them. Furthermore, you can use the website mypronouns.org [27] to learn more about how to talk about pronouns. Knowing names and pronouns may seem basic, but taking time to do this correctly can make the difference for someone feeling seen, respected, and valued in the space. We want diversity in our labs and must be ready to respond to it welcomingly. To learn more about supporting diverse identities, see 10 simple rules for building an anti-racist lab [14] and 10 simple rules for successfully supporting first-generation/low-income students in STEM [15].

### Rule 3: Proactively engage with your lab members

We’ve heard stories over the years about different lab experiences and what it is like to be in a lab where the PI breezes by everyone and goes straight to their office to handle their work for the day. Understandably, many PIs are busy; but when this happens, lab members are likely to feel unimportant to the leader of the lab. In fact, research has found that feeling ignored at work has detrimental effects on one’s physical and mental health [28]. Not being acknowledged can also have a negative impact on one’s ability to feel a sense of belonging within the lab.

We have found that many PIs rely on “open-door” policies as an engagement strategy, trusting that if any lab members need to speak with the PI, they will go to the PI’s office (thus justifying the breeze-by entrance). Given the power dynamics that exist within traditional research teams, we can think of open-door policies as a *passive* type of engagement, which places the onus of communication on research trainees, technicians, assistants, and associates when this effort should be driven *proactively* by the leaders of the space. Greeting people when entering the lab for the day can be a simple yet impactful strategy to show people that they are important and worthy of your time. Greeting your team can strengthen trust among lab members, creating a more inclusive and engaging environment. Whatever your strategy is, you should consider the inevitable power dynamics within your lab and proactively create accessible and engaging opportunities for your lab members to communicate with you and each other.

Engaging with lab members on a consistent basis normalizes and invites communication and thought exchange. In addition to serving the research program better, this strategy helps set the foundation for communicating conflict in the future. Active involvement allows you to witness practices and behaviors that can be proactively addressed to prevent major conflict.

### Rule 4: Discuss, document, and embody lab values

Lab leaders can leverage the group’s shared value of scientific mission as a starting point to highlight commonalities while building a values-driven scientific research community. Research shows us that because sense of belonging is closely tied to one’s social identities, this alone is not enough [29]. Our values are often informed by our lived experiences, our identities, and the environments in which we inhabit. Keep this in mind as you begin to discuss values.

Ideally, a discussion about the lab’s guiding values should be had during meetings when everyone comes together for the first time (i.e., the beginning of the academic year when there are likely new members in the research group), but it’s never too late to start these conversations. It could be a writing activity or an open discussion where you ask others: What do you value as a scientist? What do you value in this lab community? Or, you can use the Value Sort Tool [27] for ideas on how to start. This activity may feel uncomfortable at first, but it is important to discuss and can inform the type of lab culture you are actively creating.

Discussing values will demonstrate to the lab that you are interested in what your lab members value and how they want to shape the space. Having this discussion can create a sense of psychological safety in the lab where there are judgement-free and supportive relationships between peers and mentors [30]. It also takes the pressure off of individuals who wish to bring up challenges they are experiencing when they would otherwise have little to no foundation to bring up vulnerable topics. Creating space to discuss values will build feelings of belonging as members are personally contributing to the group, an important strategy in creating a sense of belonging.

Once norms and values have been documented, a great next step is to document what the group has collectively established. One way of doing this is through visual representations (see, for example, Sammy Katta’s work on “In this lab we believe”; [27]). Another option is to document the values of the lab through a lab contract. After your group has co-constructed a set of values that you wish to collectively uphold in the lab, you can draft a document so everyone can have a record of what was agreed upon. This will help keep accountability among group members as well as provide a values-based foundation for the lab community. It can also provide a structure for future lab meetings (i.e., we value gratitude so we begin lab meetings with kudos).

Now that values have been documented, PIs and other senior lab members can model them. Modeling the group’s values will make the active participation and shaping of the space more inviting and accessible for all lab members. This behavior sets an important example and empowers all lab members to embody lab values. Embodying the lab’s values can look like acknowledging difficult moments as they occur, holding space, extending grace, and demonstrating compassion for those who are impacted.

### Rule 5: Be transparent about lab expectations

“Ambiguity begets inequality” [31]. When expectations lack transparency, students without knowledge of how your lab operates will feel left behind or left out [32]. Be clear about rules and procedures. It is important to be clear about what is expected from each lab member. Transparent lab norms ensure everyone is on the same page. The Center for Improvement of Mentored Experiences in Research (CIMER) recommends that expectations be articulated in lab guidelines or contracts. These can be living documents that evolve over time and include information like recommended modes of communication (email, Slack, etc.), required meetings (lab meetings, journal clubs, annual retreats, conferences, etc.), how to report vacations and time away, and lists of locations of shared resources (proposal libraries, lab equipment, etc.). Importantly, set clear guidelines around what steps lab members should take to find answers when they feel confused or when something is unclear. That way, any unintentional ambiguity is addressed quickly, leading to ultimate transparency and, consequently, stronger sense of belonging in your lab.

### Rule 6: Provide opportunities to learn about each other

Every lab member is unique with their own cultural blend of traditions, values, and practices [33]. Learning about each other provides opportunities to find connection among each other and to build a sense of belonging among all members. Even something small, like having the same favorite color, can increase a sense of belonging [7]. Some labs create a lab website that has information about each lab member and provides an opportunity for individuals to carve out their unique contributions to the lab community. A lab website is a great strategy to demonstrate inclusion and belonging, but if this is something your lab does, make sure you are taking the time to keep the website updated as new members come and go and lab members’ information will likely change over time.

Alternatively, some labs may choose to learn about each other through storytelling. In the Gracia Lab at the University of Illinois at Urbana-Champaign, for example, the PI assigns The Storytelling Project [27]. For this project, each lab member tells a story that reflects their upbringing, culture, or educational journey. They choose any medium (essay, poem, song, painting) and present it to their lab group. Storytelling is a very powerful tool as it humanizes our experiences and enhances social bonds [34]. Storytelling brings people together and allows groups to find cultural matches that can support a sense of belonging and teamwork in the lab. Additionally, storytelling provides individuals an opportunity to talk about the groups’ social identities (race, ethnicity, gender, socioeconomic background) discussed in Rule 2 and how they shape their experiences in the lab. As we learn more about each other, we understand more fully how to respond to and support needs.

Another option is to take turns bringing food to lab meetings. You can encourage lab members to bring a dish that has a story behind it, such as a family cuisine, or a dish that holds a special meaning to them. This can allow lab members to learn more about each other and bond over food. However, this option might not be accessible to everyone because of dietary needs or financial constraints, so you should discuss whether or not this is something your lab wants to do. Another idea is to ask group members to share a photo (it can be a person, place, or thing) and describe why that image is important to them.

Note: It is also important to respect lab members who value their privacy. Respect their boundaries. Not being judged for their want or need of privacy, and still being included and valued, will help them feel like they belong.

### Rule 7: Foster connection outside of the lab

Like Rule 6, fostering connection outside of the lab provides lab members a chance to learn more about each other and strengthen social ties within the lab community. As a leader, you can take a proactive approach to fostering connection by hosting events outside of the lab. Here are some things you should be mindful of when planning events outside of the lab: lab members who are parents or caretakers may have more challenging schedules; hosting happy hours might exclude lab members who do not drink alcohol; activities such as golf or football may not be seen as accessible or interesting activities for all lab members.

We recommend planning your event in an inclusive way. For example, to ease the burden on parents and caretakers, encourage lab members to bring their families, partners, or someone that is close to them. You can keep it budget friendly by meeting at a park or open space on campus and inviting everyone to bring a dish—potluck style. Consider varying the time of events so that lab members with recurring commitments can be included as well. Bringing people together outside of the lab will provide an opportunity to get to know lab members by learning more about who they are outside of their research.

Note: Again, it is important to respect lab members who have boundaries and want to separate work life and personal life. Not being judged for their want or need of such boundaries, and still being included and valued, will help them feel like they belong. However, if the lack of engagement is due to lab members feeling like they can’t participate, or you notice that the typical attendees represent a particular demographic, then you’ll need to reassess the accessibility of the activities such that everyone in your lab feels comfortable attending. Many networking opportunities happen outside the confines of work, so these exclusionary acts can lead to disparate career outcomes among members of the same research group.

### Rule 8: Build in time for kudos

People feel valued when their work is recognized [35]. Recognizing individuals for their contributions and accomplishments is a great way to grow a sense of belonging among members of the lab. You can build this practice into lab meetings by opening or closing with kudos. This builds the norm of valuing contributions big and small and helps lab members stay informed about what others are working on. This can easily go sideways and make people feel excluded and undervalued if you only recognize individuals for their perceived larger accomplishments. For example, all publications should be recognized, not just those that make it into high-impact journals. Non-publication accomplishments should be given kudos as well. As a solution, you can open the meeting with: what kudos do you have to share today? Or, you can institute a practice where everyone presents something of which they are proud or you can use your morning check-ins from Rule 3 to learn more about what your lab members are doing, which can provide you with information in the kudos you give. For example, walking into the lab, you ask one of your doctoral students (we’ll call her Cheryl) how she’s doing, and she responds with information about her dissertation proposal. You can then give kudos based on what you learn. You can say, for example, “I want to give kudos to Cheryl for passing her dissertation proposal yesterday. I know she worked really hard on it and we’re all so proud of her huge milestone.” This is one way to show that members are valued in the lab space, thus strengthening their sense of belonging.

Note: Recognition is a way for the STEM identity of an individual to be nurtured [29]. Therefore, if the kudos activity becomes negative or competitive, this could be considered a reflection of the lab’s climate, culture, and values; in which case, lab leaders should take inventory on what is contributing to the scarcity mindset.

### Rule 9: Conduct equity checks

Like with many scientific processes, it is important to build in check-ins to gauge and sustain good performance. Equity checks provide an opportunity to embody values (Rule 4) and establish an actionable expectation (Rule 5). Psychological safety is key when conducting meaningful equity checks. Equity checks help to ensure lab members feel as equally valuable as others in the lab. Equity checks assess whether current lab practices are fair, whether any lab member needs some temporary assistance, or whether a particular lab member has been going “above and beyond” and would benefit from tasks being redistributed. It is important to examine if lab jobs, such as making buffers or cleaning common spaces, are equitability distributed among group members too. You might consider creating rotating jobs or annual cleanup days. Equity checks can include “opportunities for individuals to share whether they feel free to express their opinion and whether the climate of the group is hospitable to discussion and respectful disagreement” [36]. You can facilitate an equity check by asking your group: How are people feeling about the fairness of lab practices? Who needs assistance? What areas need to be revisited? These questions can start the conversation about equity in the lab and can also demonstrate that the lab truly values equity by practicing it. To learn more about the definitions of terms used in this paper, see Glossary of Terms (S2 Fig) or UNC’s professional development curriculum on Equity versus Equality, Diversity versus Inclusion [37].

In conducting equity checks, you need to be ready and equipped to respond to inequity. For example, what if lab members consistently report inequitable practices and behaviors? What if they are reporting a hostile work environment? Belonging can’t be cultivated in a space that consistently ignores incidents and leaves them unresolved. It is extremely important that the PI be willing to admit that there is a need for conflict management and to find staff members with conflict resolution expertise if the PI does not have it.

### Rule 10: Ask for feedback regularly

A gap in perception might make it difficult for PIs and senior lab members to gauge how junior lab members are feeling [38]. A study of 3,200 scientists found that there is a “perception gap” in lab experiences where senior lab members had a more positive perception of lab experiences, which differed from that of junior lab members [38]. You can mitigate this gap in perception by asking for feedback regularly. Asking for feedback is a common management and leadership practice and can make you a more effective leader [39,40]. It signals to lab members that their experiences are valued and you are interested in understanding them. Feeling valued by leadership is one way to increase the sense of belonging of members in the lab environment.

Facilitating equity checks (Rule 9) is one example of how to gather feedback from your group, but feedback can be gathered about different aspects of the research group through varied methods such as surveys and informal check-ins. Consider ways you can proactively provide monthly opportunities for lab members to give feedback. One way to do this is to create an anonymous survey where lab members can indicate how they are feeling about topics such as your leadership, inclusivity, professional development, sense of community, feeling supported in research, etc. This will provide you with more insight into how others are experiencing your leadership in the lab and give you a better understanding of how to gauge lab members’ sense of belonging. For resources on asking for feedback, see How Leaders Can Get Honest, Productive Feedback [40]. You might not always get it right the first time and that’s okay. Mistakes can be lessons learned. What’s important is that you are actively seeking feedback and looking for ways to improve your lab practices.

Note: Anonymity is a privilege that small groups might not have when providing feedback. This may not be an issue when the feedback provided will be accepted with gratitude and respect. However, retaliation is a legitimate fear among trainees, especially due to power dynamics. In these cases, faculty would benefit from professional coaching offered by universities on how to receive feedback, and perhaps a coach or other staff member can sit in on these feedback sessions to make sure they are done well. If your university does not offer coaching, consider checking with your department head or human resources to see what types of support are available.

## Conclusions

The ten simple rules listed above are practices for growing a sense of belonging in your research lab. Remember that evaluation and iteration are key to maintaining a strong sense of belonging. Consider monthly, quarterly, and annual evaluations of your team members’ feelings of belonging. It’s okay to reevaluate at any point. Your adaptability and responsiveness will prove key to developing optimal learning conditions. With some sustained effort, you will find that you’ve created a unique set of practices that foster a sense of belonging among all team members in your specific research group.

When we feel like we belong, we become positioned to do our best work. Creating and maintaining a strong sense of belonging thus benefits individuals and teams and improves the quality of science that your team produces. If done effectively, you will have an impact on the quality of the next generation of leaders in STEM who will learn from your practices.

## Supporting information

S1 Fig Summary of rules to create a sense of belonging in your research group. (TIFF)

S2 Fig Glossary of terms: Ten simple rules for creating a sense of belonging in your research group. (TIFF)
