# Supplementary figures and images for: Ten simple rules for creating a sense of belonging in your research group
Source: PLoS Comput Biol. 2022 Dec 8;18(12):e1010688. doi: 10.1371/journal.pcbi.1010688 (PMC9731414; doi:10.1371/journal.pcbi.1010688)

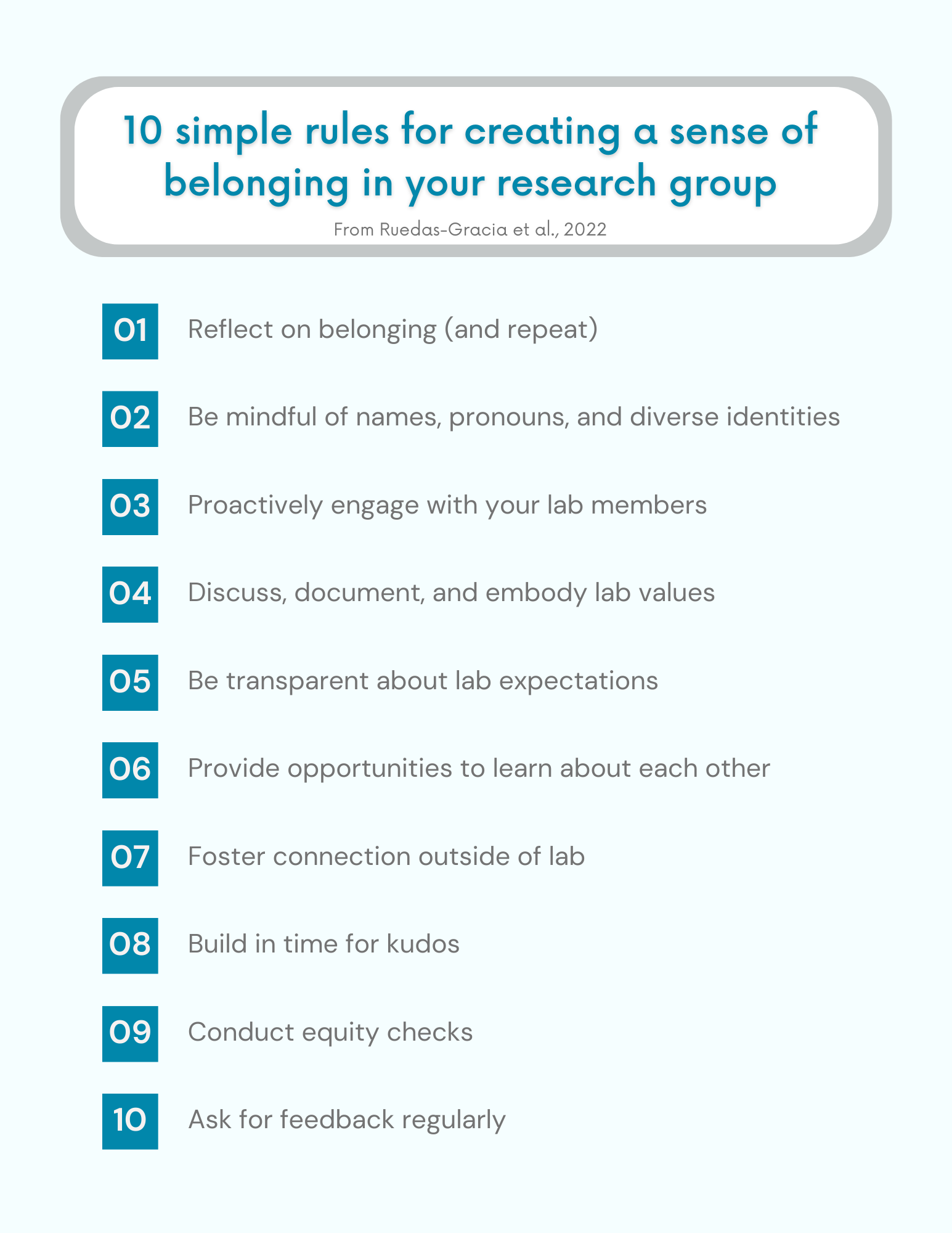

Supplement: S1 Fig — (TIFF) [file pcbi.1010688.s001.tiff]

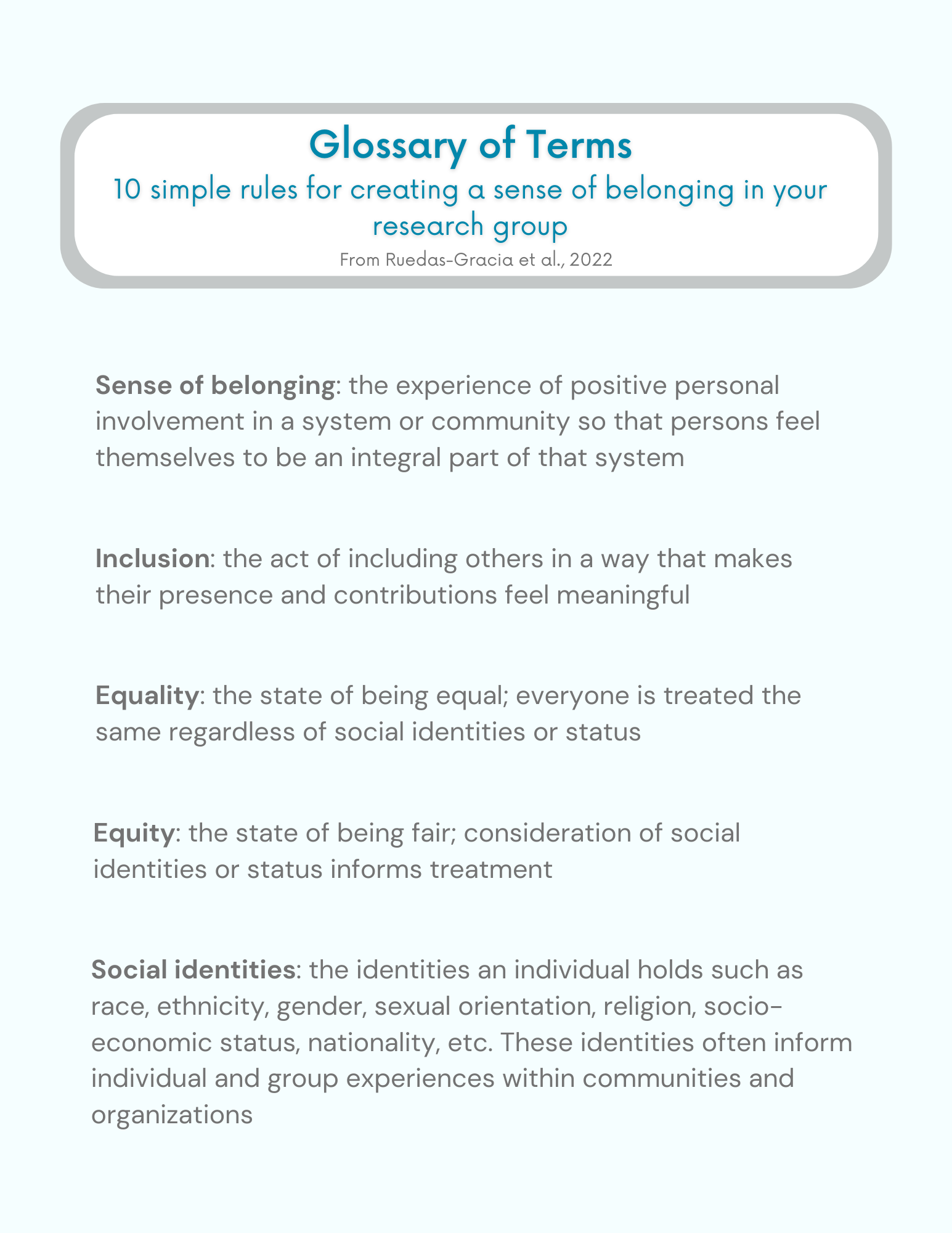

Supplement: S2 Fig — (TIFF) [file pcbi.1010688.s002.tiff]
